# DockerBIO: web application for efficient use of bioinformatics Docker images

**DOI:** 10.7717/peerj.5954

**Published:** 2018-11-27

**Authors:** ChangHyuk Kwon, Jason Kim, Jaegyoon Ahn

**Affiliations:** 1 Department of Computer Science and Engineering, Incheon National University, Incheon, The Republic of Korea; 2 MyGenomeBox, Co, Incheon, The Republic of Korea

**Keywords:** Docker, NGS pipeline, Dockerbio, RNA-Seq, DNA-Seq, RNA pipeline, DNA pipeline, Bioinformatics, Mygenomebox

## Abstract

**Background and Objective:**

Docker is a light containerization program that shows almost the same performance as a local environment. Recently, many bioinformatics tools have been distributed as Docker images that include complex settings such as libraries, configurations, and data if needed, as well as the actual tools. Users can simply download and run them without making the effort to compile and configure them, and can obtain reproducible results. In spite of these advantages, several problems remain. First, there is a lack of clear standards for distribution of Docker images, and the Docker Hub often provides multiple images with the same objective but different uses. For these reasons, it can be difficult for users to learn how to select and use them. Second, Docker images are often not suitable as a component of a pipeline, because many of them include big data. Moreover, a group of users can have difficulties when sharing a pipeline composed of Docker images. Users of a group may modify scripts or use different versions of the data, which causes inconsistent results.

**Methods and Results:**

To handle the problems described above, we developed a Java web application, DockerBIO, which provides reliable, verified, light-weight Docker images for various bioinformatics tools and for various kinds of reference data. With DockerBIO, users can easily build a pipeline with tools and data registered at DockerBIO, and if necessary, users can easily register new tools or data. Built pipelines are registered in DockerBIO, which provides an efficient running environment for the pipelines registered at DockerBIO. This enables user groups to run their pipelines without expending much effort to copy and modify them.

## Introduction

A huge number of bioinformatics tools or pipelines have been developed and distributed; however, in many cases, using them is not so easy. The reasons for this are: (1) they were developed and tested on specific versions of different operating systems; (2) they may require additional libraries; and (3) the tools or pipelines needed for analysis of big data (such as Next Generation Sequencing (NGS) experiments) requires distributed file systems and RAID settings. For these reasons, even skilled bioinformaticians have difficulty using them, and waste too much effort making environments to run them. Another problem in using bioinformatics tools is that the results can be different for different versions of the tools, OS, compilers, interpreters, libraries, settings or data, which reduces the reproducibility of experiments.

To solve these problems, several approaches have been proposed by which to wrap bioinformatics tools and the entire experimental environment (including OS, libraries, configuration files and data) into a Docker image (Beaulieu-Jones & Greene, 2017; Di Tommaso et al., 2015). Built Docker images can be registered at Docker Hub (https://hub.docker.com/), and users can simply download and run them, without making any effort to compile or make complex settings for them, and still get reproducible results (Schulz et al., 2016; Kim, Poline & Dumas, 2018). Moreover, running a Docker image guarantees performance as good as running it in a local system (Dua, Raja & Kakadia, 2014).

However, using Docker images is still not easy because there are still no clear standards for distribution of Docker images. The Docker hub can have multiple Docker images with the same objective, but different uses. For this reason, users can have difficulty selecting the proper Docker image and using it. Moreover, it is quite common for researchers to make their own pipelines with various tools; however, Docker images are often not suitable as a component of a pipeline because many of them include large databases. Even if the pipeline is built with existing Docker images, user groups can have difficulty sharing it. Users of a group may modify scripts without notification, or use different versions of the data, which results in inconsistency of the overall results.

To address these problems, we developed DockerBIO, a Java web application for easy management and use of Docker images. DockerBIO provides widely used, already verified Docker images, so users do not have to worry about selecting the right Docker image for their purpose. Users can still register existing Docker images in Docker Hub. Because tools and data are separated in DockerBIO, Docker images can be lighter and users can download them faster. DockerBIO also provides widely used datasets, as well as upload options for custom datasets. DockerBIO provides an intuitive graphical user interface (GUI) that makes setting parameters and making custom pipelines easily. The DockerBIO package can be installed on a local server or cluster, after which it provides efficient and reusable environments in which user groups can run pipelines.

A number of web-based or Docker-based platforms (such as Galaxy (Giardine et al., 2005), BioContainers (Da Veiga Leprevost et al., 2017), and RUbioSeq+ (Rubio-Camarillo et al., 2017)) have been proposed for easy biomedical analysis. In addition, the means for efficient installation or management of bioinformatics tools have been proposed, such as Bioconda (Grüning et al., 2018). However, DockerBIO has many advantages over these earlier efforts, as shown in Table 1. It is relatively easy to install DockerBIO on local servers or on a PC compared to Galaxy, which requires local installation for heavy jobs. DockerBIO provides an easy-to-use GUI and the flexibility to support any application and data.

**Table 1:** Pros and cons of existing platforms for biomedical data analysis.

	Pros (characteristics)	Cons
DockerBIO	– Easy installation on local environment – Provides easy-to-use GUI – Easy to analyze NGS data	– No community for support yet
Galaxy (Giardine et al., 2005)	– Provides easy-to-use GUI – Good community for support	– Slow running time on hosted servers – Relatively difficult to install on local servers
BioContainers (Da Veiga Leprevost et al., 2017)	– Framework or infrastructure for software standardization	– GUI is not provided – Pre-registered data is not provided
RUbioSeq+ (Rubio-Camarillo et al., 2017)	– Automated and parallelized workflows to analyze NGS data	– Limited to NGS data analysis
Bioconda (Grüning et al., 2018)	– Provides efficient way to install and manage most of bioinformatics tools	– GUI is not provided

## Methods

**Figure 1:**
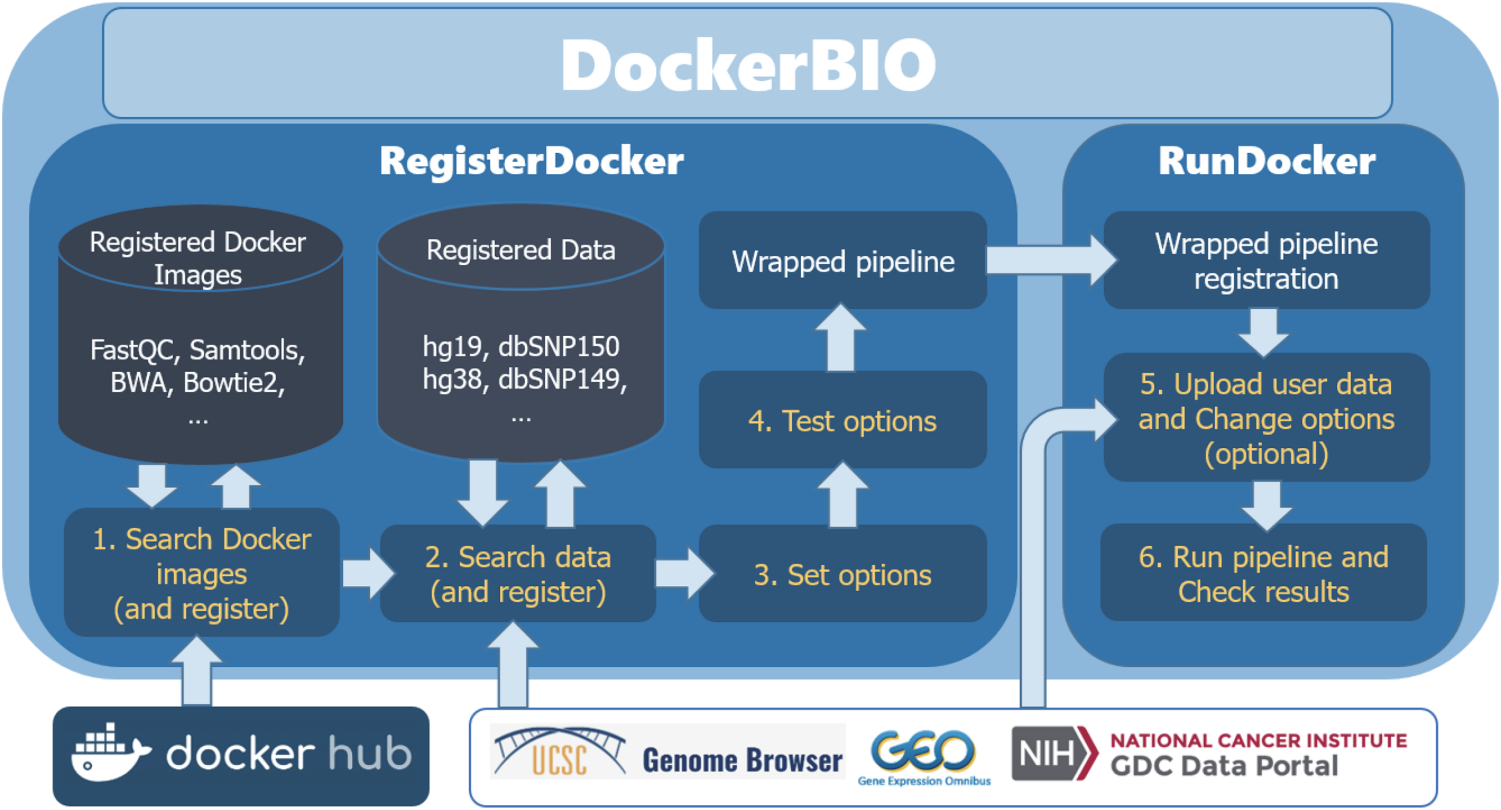
Overview of the workflow. DockerBIO is composed of RegisterDocker and RunDocker. In RegisterDocker, users can use Docker images registered in DockerBIO, or search Docker images from Docker hub. They can also use data registered in DockerBIO, or search data from other data repositories. After the options are set and tested, a pipeline is made and registered to DockerBIO in RunDocker. In RunDocker, users can upload their own data, change options, run the registered pipeline and check results.

DockerBIO is composed of *RegisterDocker*, which is used to search, edit, and register Docker images, and *RunDocker*, which is used to run Docker images. An overview of the workflow is shown in Fig. 1. Briefly, users can use DockerBIO by following these steps: (1) search needed applications (Docker images) already registered to DockerBIO, or register a needed Docker image (in *RegisterDocker* menu), (2) upload their own data and edit options for Docker images (in *RegisterDocker* menu), and (3) test and run a Docker image with uploaded data and options (in *RunDocker* menu).

### RegisterDocker

On the *RegisterDocker* page, users can search Docker images in the Docker Hub; then edit and register them to DockerBIO. Widely used bioinformatics tools such as FastQC, Picard, BWA and GATK, are pre-registered in DockerBIO (Table 2). Those tools are verified and light-weight Docker images without data. Users can also search Docker images already registered at the Docker Hub. Although DockerBIO is optimized for specific jobs such as aligning High-Throughput sequencing reads or calling single nucleotide polymorphisms (SNPs), it is flexible enough to run any bioinformatics tool that is provided as a Docker image. Widely used big data such as the Human Genome Reference (hg19, hg38, etc.) or dbSNP, are pre-registered in DockerBIO (Table 3), but users can also upload custom data. The list of pre-registered bioinformatics tools and data will be updated periodically.

**Table 2:** Pre-registered Dockers.

Docker name	Tool lists	Reference
netbuyer/wgs	bwa, picard, gatk	Li & Durbin (2010) and Van der Auwera et al. (2013)
netbuyer/rna_seq	hisat2, samtools, stringtie, gffcompare	Li (2011) and Pertea et al. (2016)
conradstoerker/fastqc	FastQC	Andrews (2015)
comics/bwa	BWA(mem), BWA	Li & Durbin (2010)
alexcoppe/picard	Picard(sort)	Van der Auwera et al. (2013)
comics/bowtie2	bowtie2	Langmead & Salzberg (2012)
netbuyer/rna_seq:0.1	hisat2	Pertea et al. (2016)
alexcoppe/picard	picard	Van der Auwera et al. (2013)
biocontainers/samtools	samtools	Li (2011)
biodckrdev/gatk	GATK3.5	Van der Auwera et al. (2013)
alexcoppe/snpsift	SnpSift annotate	Cingolani et al. (2012)

**Table 3:** Pre-registered datasets.

Data name	Description	Reference
Reference DNA(DNA-Seq): hg^*^19, hg38	Reference whole human genome sequence for running DNA-Seq	Shepelev et al. (2015)
Reference RNA(RNA-Seq): RNA_hg19, RNA_hg38	Reference transcriptome sequence for running RNA-Seq	
dbSNP(hg38): dbSNP141, 142, 144, 146, 147, 150	SNP identified from hg38	Speir et al. (2015)
dbSNP(hg19): dbSNP138, 141, 142, 144, 146, 147, 150	SNP identified from hg19	
Annotation RNA(hg38): RNA_hg38_annotated.gtf	Exon and intron annotations based on hg38	
Annotation RNA(hg19): RNA_hg19_annotated.gtf	Exon and intron annotations based on hg19	

**Note:**

* hg: human genome.

**Figure 2:**
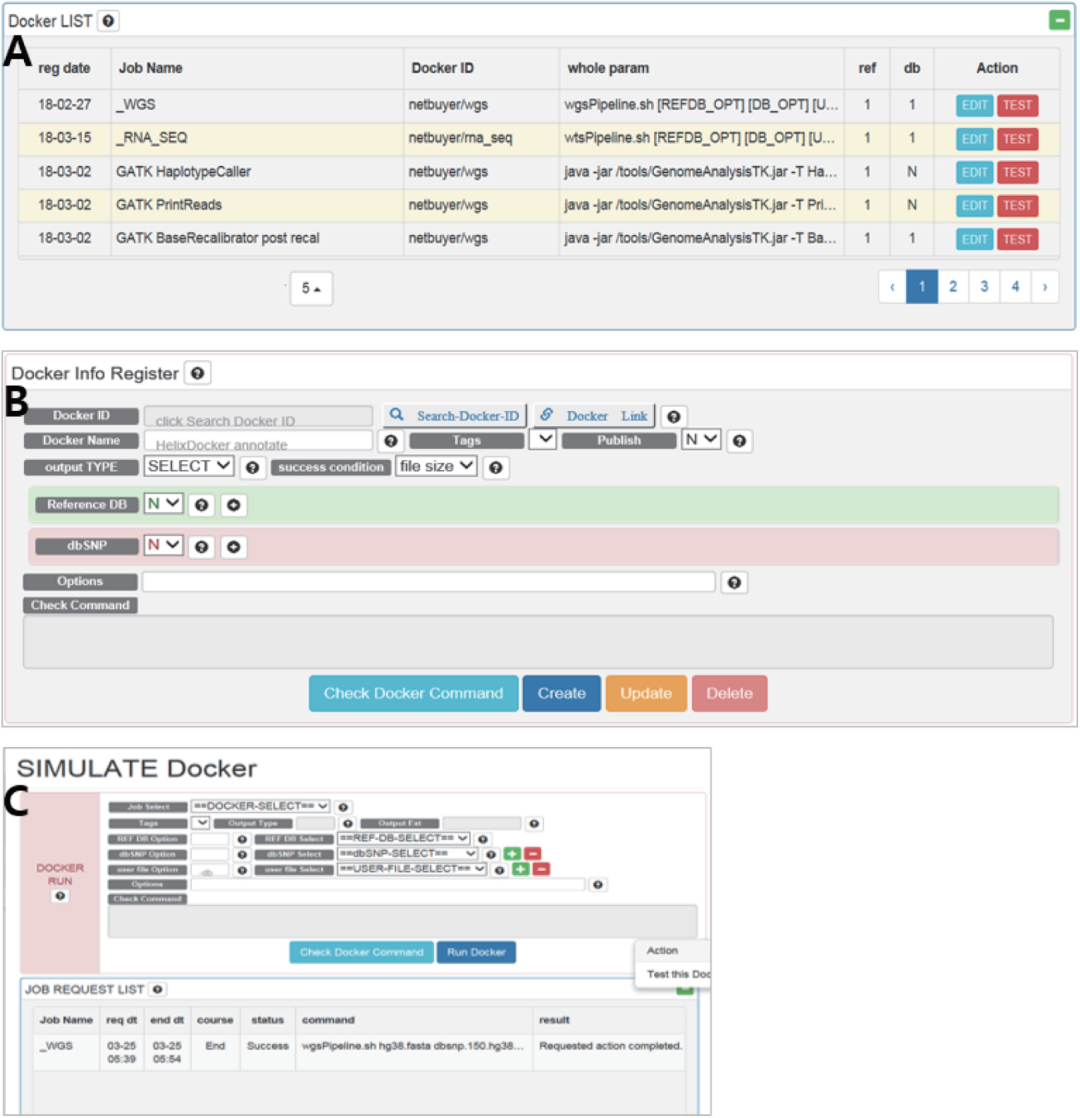
(A) Docker LIST, (B) Docker info register and (C) SIMULATE in *RegisterDocker*. (A) Docker LIST: menus for editing and testing options. (B) Docker Info Register: Menus for searching Docker images from Docker Hub, registering dataset and setting options. (C) SIMULATE: menus for testing registered Docker and options.

The *RegisterDocker* page consists of two menus: Docker LIST (Fig. 2A) and Docker Info Register (Fig. 2B). Docker LIST is a menu for editing options and for testing whether the registered option works. After setting the basic options for tools, users can test whether the registered Docker images and options are working, which is accessed through the “Test” button on the SIMULATE page (Fig. 2C).

The Docker Info Register provides menus for searching Docker images registered in Docker Hub and downloading them to the local system. Docker images can be searched using the “Search-Docker-ID” menu. Searching with the “BWA” keyword, hundreds of BWA Docker images registered in Docker Hub can be found. Users can select a desired Docker image, or a Docker image with a large number of downloads or stars if users do not know how to select an appropriate Docker image. Then users can click the “select” button to register it in DockerBIO.

DockerBIO provides a variety of datasets including human reference sequences and dbSNP databases (Table 3). By referencing the user manual, users can upload dbAll.tar.gz and refDbAll.tar.gz to DockerBIO, as well as their custom datasets. “Reference DB” and “dbSNP” are menus for selecting registered datasets. “Options” is a menu that provides additional options for a program. To configure and check all options, users can select “Check Docker Command”.

### RunDocker

**Figure 3:**
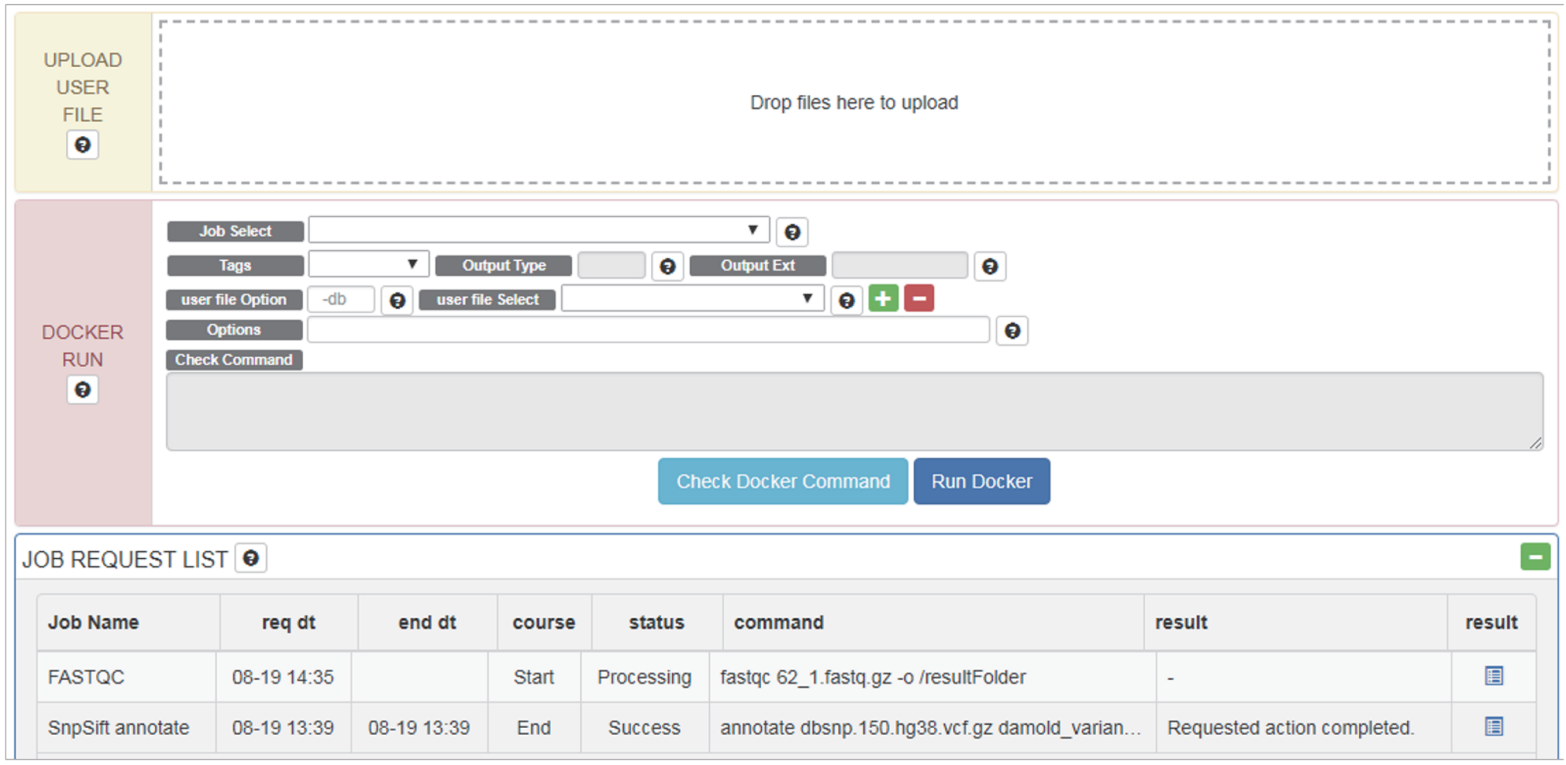
Options and menus on the *RunDocker* Page. UPLOAD USER FILE: for uploading user data files for analysis., DOCKER RUN: menus for running registered pipeline. Please refer to the UserManual for a detailed description of each command., JOB REQUEST LIST: menu for checking the result.

Once a Docker image and data are selected, users can edit commands and options intuitively through the GUI (Fig. 3). Tools, data and command (options) are wrapped into a pipeline as a Docker image and this image is tested for errors. If a test is successful, the Docker image created is also registered on the *RunDocker* page. Docker images from *RegisterDocker* can easily be run through the GUI provided on the *RunDocker* page. Users can run Docker images without any changes, or make changes to options. We also use the java spring monitoring module and mass upload spring module to enable the uploading of large data (over 100 GB) in the web page environment.

Users can upload files for analysis in “UPLOAD USER FILE” menu by drag and drop. For whole genome analysis, users should select _WGS (netbuyer/wgs) Docker from the “Job Select” menu. Then users can set DIR, LOG, or FILE, to specify results in the form of log files, specific files, or directories, respectively for “Output Type” menu, select a reference database from the “REF DB Select” menu, and then select dbSNP files in the “dbSNP Select” menu. When one end of paired-end files is uploaded, it can be checked using “user file Select” menu. Users can then select the other end of paired-end files using the plus button. Using the “Options” menu, users can modify additional options. After verifying that Docker works properly using the “Check Docker Command,” users can run Docker by pressing “Run Docker”.

### Performance trials

We constructed DNA-Seq and RNA-Seq analysis pipelines using DockerBIO, and then compared them with (1) LocalM, a local pipeline (Local Machine, all programs in a pipeline on a local machine) and (2) MultiD (Multiple Docker: each program in one Docker image, and multiple Dockers connected as a pipeline). Our pipelines are called OneD (One Docker) because the whole pipeline is one Docker image and MultiD, for simulating typical usage of multiple Docker images when they are incorporated into a pipeline. We performed two DNA-seq and two RNA-seq experiments, and the data descriptions for the experiments are provided in Table 4. Each DNA-seq and RNA-seq pipeline was run six and five times, respectively.

**Table 4:** Data description.

Experiment	Sample ID	Reference	#Reads	Description
DNA-seq1	NA12750	1000 Genome project (ftp://ftp-trace.ncbi.nlm.nih.gov/giab/ftp/release/)	11,964,008	Lymphoblastoid cell lines from the 1000 Genomes
DNA-seq2	NA12878	GIAB (ftp://ftp-trace.ncbi.nlm.nih.gov/giab/ftp/release/)	28,991,397	Extracted by random sampling using a 300× high depth file of NA12878.
RNA-seq1	SRX1952336	GEO (https://www.ncbi.nlm.nih.gov/sra/SRX1952336)	36,313,342	Transcriptomic differences associated with TSC2 Gene expression loss in Lymphangioleiomyomatosis (human cells)
RNA-seq2	GSM3244545	GEO (https://www.ncbi.nlm.nih.gov/geo/query/acc.cgi?acc=GSM3244545)	129,881,552	Role of AHR in lymphoblastoid cell lines.

For the experiments, we used BWA for alignment, Picard for sorting SAM, GATK for variant callers, and Stringtie for assembling RNA-seq alignments. Although many DNA-seq and RNA-seq read aligners that outperform BWA have been developed recently, we used BWA because it is still a widely used aligner. Likewise, we used Picard, GATK and Stringtie, because they are essentially standards for DNA-seq or RNA-seq data analysis.

## Results

The running times for two DNA-seq experiments are shown in Tables 5 and 6, and for two RNA-seq experiments in Tables 7 and 8. Detailed experimental results are provided in Supplementary Tables 1 and 2. To compare the running times of OneD, MultiD and LocalM (java1.7 for DNA-seq), we performed ANOVA test. The *p*-values to reject the null hypothesis that running times are not different for DNA-seq1, DNA-seq2, RNA-seq1 and RNA-seq2 were all greater than 0.05 (0.9546, 0.0542, 0.8963 and 0.9425, respectively), so we concluded that there was no significant difference and that DockerBIO did not slow down performance. OneD showed better performance than MultiD, probably due to overheads in the CPU and memory related to switching Docker images. We also confirmed that the use of GATK for DNA-Seq pipelines involved a considerable time difference related to the java version. The default version of Java is 1.8, but GATK 3.5 requires 1.7. We have confirmed that gatk3.x Docker images, which are distributed in Docker Hub, use a lot of Java’s basic 1.8. DockerBIO effectively provides the proper environment for tools, so it eliminates the tiring processes previously needed to set up such environments.

**Table 5:** Running time for DNA-seq1 (average of six run times).

	Average running time (hh:mm:ss)			
	OneD	MultiD	LocalM (java1.7)	LocalM (java1.8)
Alignment	0:27:53 (±3)	0:27:24 (±62)	0:27:18 (±35)	0:27:20 (±25)
Sorting	0:04:57 (±12)	0:04:26 (±13)	0:04:32 (±18)	0:04:32 (±18)
RemoveDuplicate	0:05:57 (±29)	0:05:20 (±6)	0:05:50 (±19)	0:05:48 (±20)
BaseRecal	0:19:31 (±62)	0:19:12 (±46)	0:19:12 (±62)	0:19:11 (±62)
BaseRecal_post	0:25:53 (±4)	0:26:22 (±23)	0:26:05 (±34)	0:26:33 (±53)
PrintReads	0:12:55 (±3)	0:13:20 (±13)	0:14:10 (±11)	0:14:18 (±17)
HaplotypeCaller	2:13:56 (±42)	2:15:17 (±28)	2:13:36 (±252)	2:52:05 (±498)
Mean overall time	3:51:02 (±123)	3:51:22 (±152)	3:50:43 (±335)	4:29:47 (±526)

**Notes:**

1. 11,964,008 DNA-seq reads from NA12750.

2. Number in parenthesis is a standard deviation in seconds.

3. Alignment: BWA alignment, Sorting: Picard sorting, BaseRecal: GATK BaseRecalibrator, BaseRecal_post: GATK BaseRecalibrator second step.

4. LocalM: Local Machine implemented a local pipeline., MultiD: Multiple Docker, multiple Dockers connected as a pipeline., OneD: One Docker implemented whole pipeline is one Docker image.

**Table 6:** Running time for DNA-seq2 (average of six run times).

	Average running time (hh:mm:ss)			
	OneD	MultiD	LocalM (java1.7)	LocalM (java1.8)
Alignment	2:26:00 (±212)	2:24:45 (±180)	2:24:53 (±130)	2:26:47 (±326)
Sorting	0:28:16 (±49)	0:28:59 (±71)	0:28:14 (±50)	0:28:05 (±35)
RemoveDuplicate	0:26:14 (±58)	0:25:40 (±19)	0:25:29 (±40)	0:25:22 (±53)
BaseRecal	1:55:24 (±95)	2:05:48 (±71)	1:54:05 (±87)	1:54:12 (±44)
BaseRecal_post	2:57:38 (±2,292)	3:25:45 (±1,867)	3:11:06 (±1,348)	3:29:24 (±746)
PrintReads	2:16:45 (±48)	2:14:23 (±75)	2:19:04 (±108)	2:18:50 (±116)
HaplotypeCaller	4:41:57 (±43)	4:55:41 (±125)	4:40:11 (±81)	7:38:42 (±79)
Mean overall time	15:12:15 (±2,473)	16:01:01 (±1,989)	15:23:01 (±1,364)	18:41:23 (±1,024)

**Notes:**

1. 28,991,397 DNA-seq reads from NA12878.

2. Number in parenthesis is a standard deviation in seconds.

3. Alignment: BWA alignment, Sorting: Picard sorting, BaseRecal: GATK BaseRecalibrator, BaseRecal_post: GATK BaseRecalibrator second step.

4. LocalM, Local Machine implemented a local pipeline; MultiD, Multiple Docker, multiple Dockers connected as a pipeline; OneD, One Docker implemented whole pipeline is one Docker image.

**Table 7:** Running time for RNA-seq1 (average of five run times).

	Average running time (hh:mm:ss)		
	OneD	MultiD	LocalM
Alignment	0:21:02 (±117)	0:21:10 (±119)	0:20:52 (±118)
Sorting	0:13:35 (±82)	0:14:42 (±90)	0:14:16 (±81)
ST_gtf	0:06:29 (±35)	0:06:29 (±37)	0:06:08 (±36)
ST_Mer_gtf	0:00:11 (±1)	0:00:11 (±1)	0:00:07 (±1)
GT_Mer_gtf	0:00:14 (±2)	0:00:14 (±2)	0:00:19 (±2)
BG_Mer_gtf	0:08:58 (±49)	0:08:58 (±50)	0:08:48 (±50)
Mean overall time	0:50:29 (±283)	0 :51:44 (±293)	0:50:31 (±283)

**Notes:**

1. 36,313,342 RNA-seq reads from SRX1952336.

2. Number in parenthesis is a standard deviation in seconds.

3. Alignment: BWA alignment, Sorting: Picard sorting, ST_gtf: Stringtie gtf generation step, ST_Mer_gtf: Stringtie merge step, GT_Mer_gtf: gffcompare step, BG_Mer_gtf: BallGown step.

4. LocalM, Local Machine implemented a local pipeline; MultiD, Multiple Docker, multiple Dockers connected as a pipeline; OneD, One Docker implemented whole pipeline is one Docker image.

**Table 8:** Running time for RNA-seq2 (average of five run times).

	Average running time (hh:mm:ss)		
	OneD	MultiD	LocalM
Alignment	46:42 (±34)	46:20 (±21)	47:39 (±32)
Sorting	44:16 (±69)	44:18 (±70)	42:08 (±61)
ST_gtf	05:55 (±4)	05:48 (±2)	06:26 (±17)
ST_Mer_gtf	00:12 (±1)	00:11 (±0)	00:13 (±1)
GT_Mer_gtf	00:16 (±0)	00:16 (±0)	00:16 (±0)
BG_Mer_gtf	05:32 (±2)	05:27 (±3)	05:35 (±2)
Mean overall time	1:42:53 (±100)	1:42:21 (±78)	1:42:18 (±66)

**Notes:**

1. 129,811,552 RNA-seq reads from GSM3244545.

2. Number in parenthesis is a standard deviation in seconds.

3. Alignment: BWA alignment, Sorting: Picard sorting, ST_gtf: Stringtie gtf generation step, ST_Mer_gtf: Stringtie merge step, GT_Mer_gtf: gffcompare step, BG_Mer_gtf : BallGown step.

4. LocalM, Local Machine implemented a local pipeline; MultiD, Multiple Docker, multiple Dockers connected as a pipeline; OneD, One Docker implemented whole pipeline is one Docker image.

Tables 5–8 shows that the bioinformatics tools used for the experiments, regardless of the OS and language in which they were developed, require similar running time when they are run in a local machine, as single Docker and as multiple Dockers. The more important factor for performance of Docker images is selection of the proper version of OS or programming language.

## Discussion

We tested DockerBIO using DNA-seq and RNA-seq pipelines, but we think DockerBIO may be useful for a wider range of researchers active in various subfields, such as ecology, evolutionary biology, structural biology, systems biology, and so on, because DockerBIO provides a flexible environment that can accommodate a variety of bioinformatics tools.

DockerBIO was tested on Linux and Mac OS, but the Windows environment is not supported because of hyper-v setting issues. To use DockerBIO in a Windows environment, using the Docker Toolbox or deactivating the hyper-v option in the BIOS is recommended.

## Conclusions

In this paper, we propose DockerBIO, a Java web application that provides an environment for easier bioinformatic analysis. By default, DockerBIO provides many well-known bioinformatics programs and data for DNA-seq and RNA-seq analysis. However, users can also run any program with custom data. Moreover, users can easily build a pipeline with registered tools and data using DockerBIO. In this paper, we report that the performance of a pipeline that contains several Docker images used for NGS analysis was not degraded (Tables 5–8).

Users can easily run bioinformatics tools or pipelines without making any effort to compile or install them, and also do not have to worry about the proper running environment. DockerBIO provides an efficient GUI for test and step-by-step running environments, as well as the best environment for group users to run similar pipelines without expending much effort to copy and modify them.

## Supplemental Information

10.7717/peerj.5954/supp-1 Supplemental Information 1 Run times for DNA-Seq experiments.

10.7717/peerj.5954/supp-2 Supplemental Information 2 Run times for RNA-Seq experiments.

## Additional Information and Declarations

### Data Availability

The following information was supplied regarding data availability: http://dockerbio.mygenomebox.com:8080/html/source.zip DockerBIO: https://github.com/narrowpath/DockerBIO UCSC human sequence: http://hgdownload.cse.ucsc.edu/downloads.html#human.
